# X-Ray Dark-field Imaging to Depict Acute Lung Inflammation in Mice

**DOI:** 10.1038/s41598-018-20193-8

**Published:** 2018-02-01

**Authors:** Katharina Hellbach, Felix G. Meinel, Thomas M. Conlon, Konstantin Willer, Andre Yaroshenko, Astrid Velroyen, Margarita Braunagel, Sigrid Auweter, Maximilian F. Reiser, Oliver Eickelberg, Franz Pfeiffer, Ali Ö. Yildirim

**Affiliations:** 1 Department of Radiology, University Hospital, LMU Munich, Marchioninistr.15, 81377 Munich, Germany; 2 Department of Radiology, Rostock University Medical Center, Ernst-Heydemann-Str. 6, 18057 Rostock, Germany; 3 German Center for Lung Research (DZL), Munich, Germany; 4 Comprehensive Pneumology Center, Institute of Lung Biology and Disease, Helmholtz Zentrum München, Ingolstaedter Landstrasse 1, 85764 Oberschleissheim, Germany; 5 Chair for Biomedical Physics, Department of Physics & Munich School of Bioengeneering, Technical University of Munich, James-Franck Str. 1, 85748 Garching, Germany

## Abstract

The aim of this study was to evaluate the feasibility of early stage imaging of acute lung inflammation in mice using grating-based X-ray dark-field imaging *in vivo*. Acute lung inflammation was induced in mice by orotracheal instillation of porcine pancreatic elastase. Control mice received orotracheal instillation of PBS. Mice were imaged immediately before and 1 day after the application of elastase or PBS to assess acute changes in pulmonary structure due to lung inflammation. Subsequently, 6 mice from each group were sacrificed and their lungs were lavaged and explanted for histological analysis. A further 7, 14 and 21 days later the remaining mice were imaged again. All images were acquired with a prototype grating-based small-animal scanner to generate dark-field and transmission radiographs. Lavage confirmed that mice in the experimental group had developed acute lung inflammation one day after administration of elastase. Acute lung inflammation was visible as a striking decrease in signal intensity of the pulmonary parenchyma on dark-field images at day 1. Quantitative analysis confirmed that dark-field signal intensity at day 1 was significantly lower than signal intensities measured at the remaining timepoints, confirming that acute lung inflammation can be depicted *in vivo* with dark-field radiography.

## Introduction

Acute lung inflammation is characterized by decreased endothelial cell barrier function, increased vascular permeability and influx of neutrophils leading to the development of acute bilateral, pulmonary infiltrates, followed by impaired oxygenation^1^. These pathomechanisms play an important role in the development of acute lung injury (ALI), a severe form of acute respiratory distress syndrome (ARDS) in humans. Acute lung inflammation can occur either after direct (e.g. due to pneumonia, toxic inhalation) or indirect damage of pulmonary parenchyma (e.g. trauma, sepsis)^2^. With a mortality rate of 40–50% ARDS represents one of the most deadly lung diseases^3^. About 30% of patients initially diagnosed with mild ARDS will progress to moderate and severe forms of the disease^4^, pointing out how vital early diagnosis of acute lung inflammation is. Reliable diagnosis remains challenging, as clinical signs are rather unspecific, leading to a high number of misdiagnoses^5^.

Chest radiography is still the most frequently used radiological tool to diagnose and monitor lung inflammation^6^. Radiographic findings, such as bilateral lung opacities, that can either appear diffuse, patchy or homogenous, reflect interstitial or alveolar edema^7–9^. However, as these signs are nonspecific and can occur in different kinds of pulmonary edema (e.g. due to heart failure), high interobserver variability is found in chest radiograph-based diagnosis of acute lung inflammation^10^.

Computed tomography (CT) of the lung is more accurate than chest radiography in detecting underlying causes as well as complications of acute lung response. Although CT is the gold standard in diagnosing lung inflammation, difficulties and risks of moving patients from intensive care units as well as the comparatively high radiation dose limits its use^11,12^.

X-ray dark-field projection radiography could be a potential solution to this problem as it might provide important information to pathological findings in transmission images without adding the inconveniences linked to CT.

Dark-field images are generated by implementing a three grating Talbot-Lau interferometer in between the X-ray source and detector, and moving one of the gratings perpendicular to the beam over one grating period. Applying Fourier analysis to the intensity registered in each pixel for all grating positions yields conventional transmission, differential phase-contrast and dark-field images. The dark-field image visualizes small-angle scattering of X-rays within tissue. Due to its alveolar microstructure healthy lung parenchyma generates a strong dark-field signal, which decreases in various lung diseases that affect the integrity of the alveoli. Phase-contrast images visualize the phase-shift of X-ray beams within tissue with enhanced representation of edges, making large airways such as the trachea or main bronchi visible^13–15^.

Diagnosing lung disorders is of particular interest due to the strong dark-field signal generated by pulmonary tissue. Added diagnostic value of dark-field compared to transmission imaging has been shown for various lung diseases, such as pneumothoraces^16^, emphysema^17^, fibrosis^18^ and bronchopulmonary dysplasia^19^. Based on these promising results we now aimed at investigating the value of dark-field imaging for the diagnosis of acute lung inflammation.

The purpose of the present study was to evaluate whether imaging pathological changes due to acute lung response *in vivo* is possible using this new imaging technique, to correlate observed alterations in dark-field signal intensities with histopathology as well as with signal changes in transmission images and to discuss a potential added diagnostic value of dark-field over transmission imaging.

## Results

### Elevated neutrophil recruitment into the lung and subsequent emphysema development

**Figure 1:**
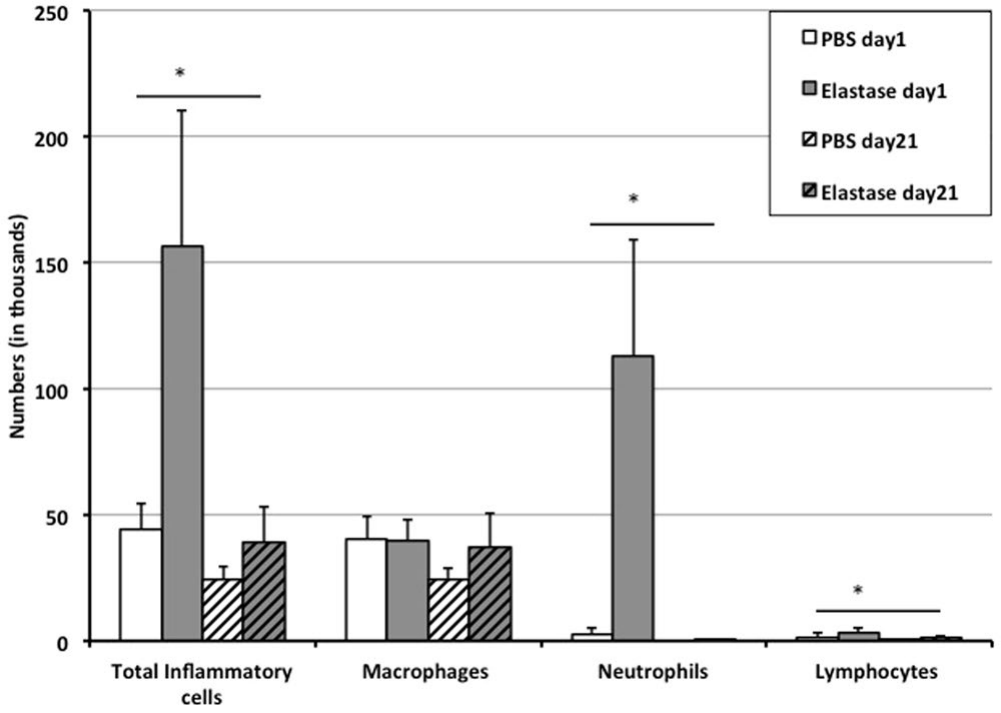
BAL cell counts. Total and differential cells counted in bronchoalveolar lavages of animals in the control group and experimental group at day 1 and day 21 after application of PBS or elastase. *p < 0.05.

To investigate the development of acute inflammatory response, we analyzed differential and total cell number in bronchoalveolar lavage (BAL) fluid and performed histology in porcine pancreatic elastase (PPE) and phosphate buffered saline (PBS) treated age-matched mice. We found that a mean number of 4.4 × 10^4^ ± 1.0 × 10^4^ leucocytes were counted in lavages of control animals, compared to a strongly increased number of 1.6 × 10^5^ ± 5.4 × 10^4^ leucocytes in animals that had received PPE the previous day (p = 0.004; Fig. 1).

No significant difference was found in numbers of macrophages (p = 0.87), whereas numbers of neutrophils and lymphocytes were significantly higher for animals in the experimental group (neutrophils: 1.1 × 10^5^ ± 4.6 × 10^4^; lymphocytes: 3.4 × 10^3^ ±1.6 × 10^3^) compared with cell counts in the control group (neutrophils: 2.4 × 10^3^ ± 2.8 × 10^3^, p < 0.004; lymphocytes: 1.4 × 10^3^ ± 1.5 × 10^3^, p = 0.04). As an additional sign of lung injury, lavages in the experimental group showed a reddish color, typical for fluid enriched with erythrocytes, compared to a clear appearance of lavages in the control group. 21 days after application of elastase the number of leucocytes had returned to baseline (3.9 × 10^4^ ± 1.4 × 10^4^; p = 0.1 compared to control group at day 21), indicating that the acute inflammatory reaction had stopped.

**Figure 2:**
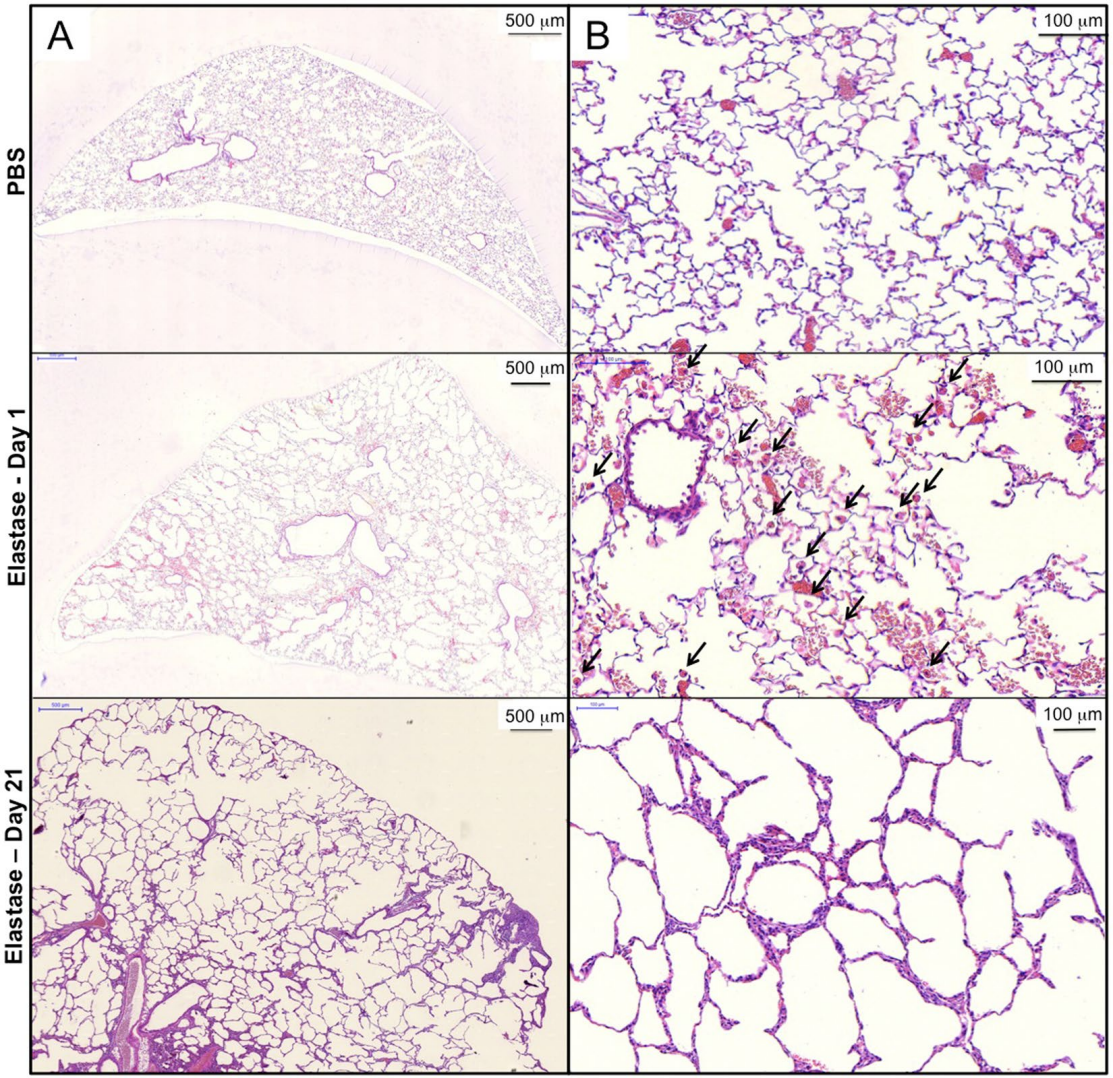
Representative H&E stained lung sections of a control mouse (upper row) and a mouse of the experimental group 1 day (middle row) and 21 days (lower row) after application of elastase. (**A**) Overview, (**B**) magnification for detailed analysis. Tissue macrophages are indicated by black arrows in (**B**).

Representative examples of haematoxylin and eosin (H&E) stained sections from control lungs and lungs surgically extracted 1 and 21 days after application of elastase are presented in Fig. 2A and B. Histologic data shows hyperemia, alveolar hemorrhage, mononuclear/neutrophilic infiltrates and numerous alveolar macrophages (black arrows) after 1 day of elastase treatment. These alterations were not found in the control lung and the lung 21 days after induction of emphysema (Fig. 2B).

**Figure 3:**
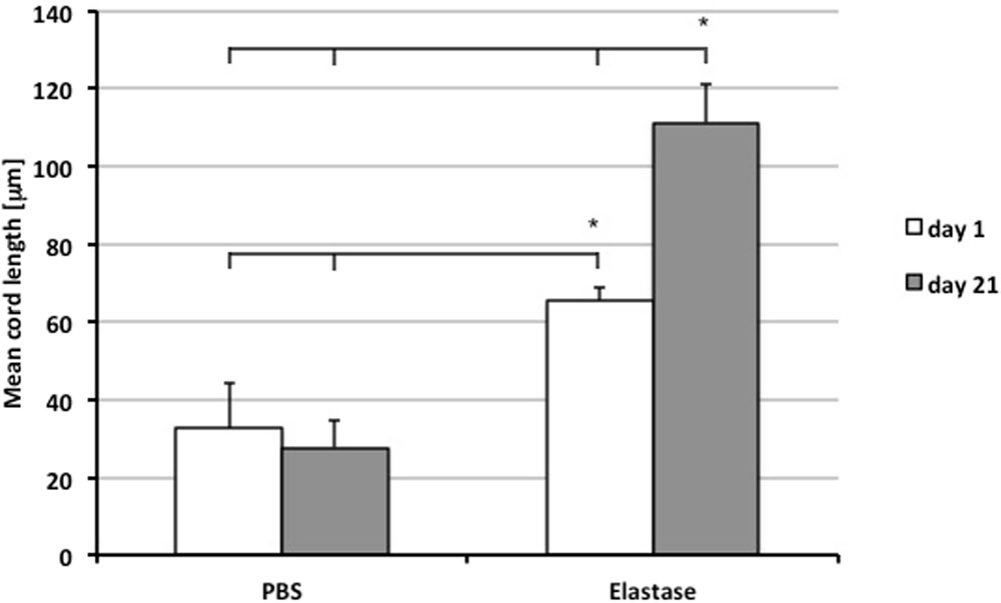
Mean cord length of animals 1 day (white bar) and 21 days (grey bar) after application of PBS or elastase. *p < 0.05.

Figure 2 also demonstrates that after only 1 day of elastase application, there is an enlargement of the airspaces, which is significantly worsened at day 21. To quantify the changes in airspace enlargement induced by elastase treatment, mean cord length (MCL) was measured using a computer assisted stereological toolbox. MCL-quantification confirmed that early pulmonary emphysema had developed one day after orotracheal application of elastase: MCL was 65.2 ± 7.1 μm in the elastase group, compared to 32.6 ± 11.7 μm in the control group (p < 0.001). 21 days after treatment with elastase, severe emphysema was found as indicated by a significantly elevated MCL level of 111.2 ± 10 μm (p < 0.001 compared to control and day 1 after application of elastase) (Fig. 3).

These results indicate that elastase induced severe acute lung inflammation and mild pulmonary emphysema one day after application, with emphysema progressively worsening over time despite a resolution of inflammation.

### Qualitative evaluation of radiographs

To evaluate the potential of X-ray dark-field imaging to image acute lung injury, we analyzed the animals *in vivo* before and after PPE treatment on days 0, 1, 7, 14 and 21.

**Figure 4:**
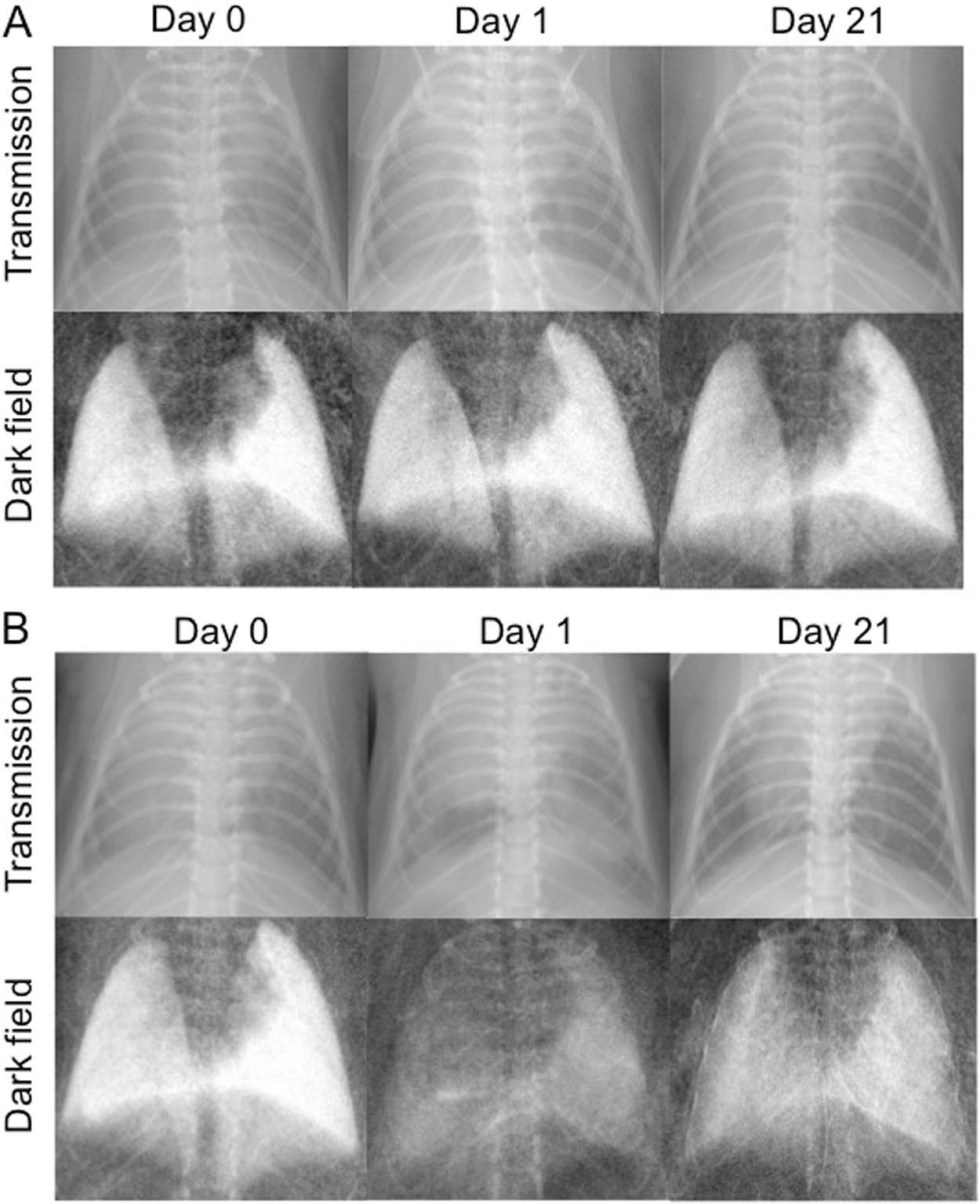
Visual example for X-ray transmission (upper row) and X-ray dark-field (lower row) images of mice before (Day 0), one day and 21 days after treatment with PBS (**A**) or elastase (**B**).

Figure 4 gives an example of two mice, one control mouse (A) that was scanned prior to PBS application and one mouse of the experimental group (B) that received elastase immediately after the first scan. Both mice were additionally imaged 1 day and 21 days after application of PBS or elastase. As shown in A, the mouse in the control group does not show any alterations in signal intensities at any point in time, neither in the transmission nor in the dark-field image. By contrast, in B changes of a mouse’s lung due to acute lung injury caused by application of elastase are revealed. Whereas the lung appears healthy at day 0, it shows a strong loss in dark-field signal intensity at day 1. Correspondingly, partially confluent opacities due to inflammation can be observed in the transmission image. After 21 days this mouse’s lung generates a slightly increased dark-field signal compared to day 1 but still shows an obvious signal loss compared to day 0. When assessing the corresponding transmission image 21 days after application of elastase only subtle, indirect signs of pulmonary emphysema are detected, such as an enlarged lung volume leading to lowered diaphragms.

### Quantitative evaluation of radiographs

**Figure 5:**
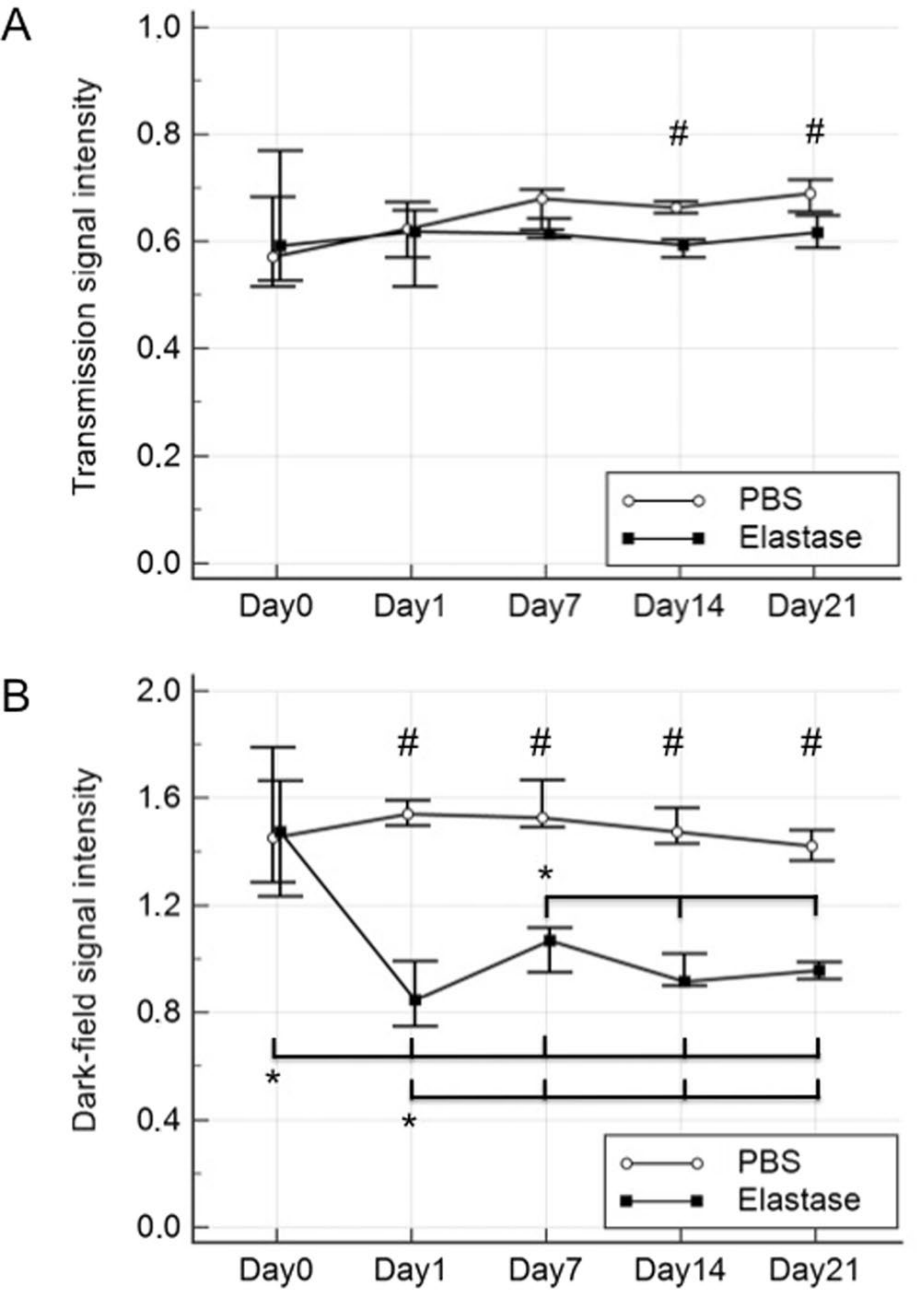
Quantitative analysis of acute lung inflammation development over time. Medians of pulmonary signal intensities measured in transmission (**A**) and dark-field (**B**) images of control (white circles) and experimental animals (black squares). Ranges are indicated by black bars. ^#^p < 0.05 at one timepoint between experimental and control group. *p < 0.05 between different timepoints within experimental or control group.

To evaluate whether alterations in lung parenchyma due to injection of PPE are quantifiable in radiographic images, the development of transmission (A) and dark-field (B) signal intensities over time (Fig. 5) was monitored.

No statistically significant change in transmission signal intensities between time points was observed within either treatment group (p > 0.5). However, a significant difference in the transmission signal was detected between the experimental and control groups at day 14 (p = 0.01) and day 21 (p = 0.01). Between time points, no changes in dark-field signal intensity was observed for the control group (p > 0.5), whereas in the experimental group signal intensity was significantly lower at day 1 compared to all other timepoints (p < 0.5). From day 1 on, dark-field signal intensities were significantly lower compared to day 0 (p < 0.5). There was no statistically significant difference between the dark-field signal intensities of days 14 and 21 within the experimental group. Between both groups, a significant difference in the dark-field signal intensity was measured at day 1 (p = 0.002) as well as days 7, 14 and 21 (p = 0.01 each).

### Quantitative histogram evaluation

**Figure 6:**
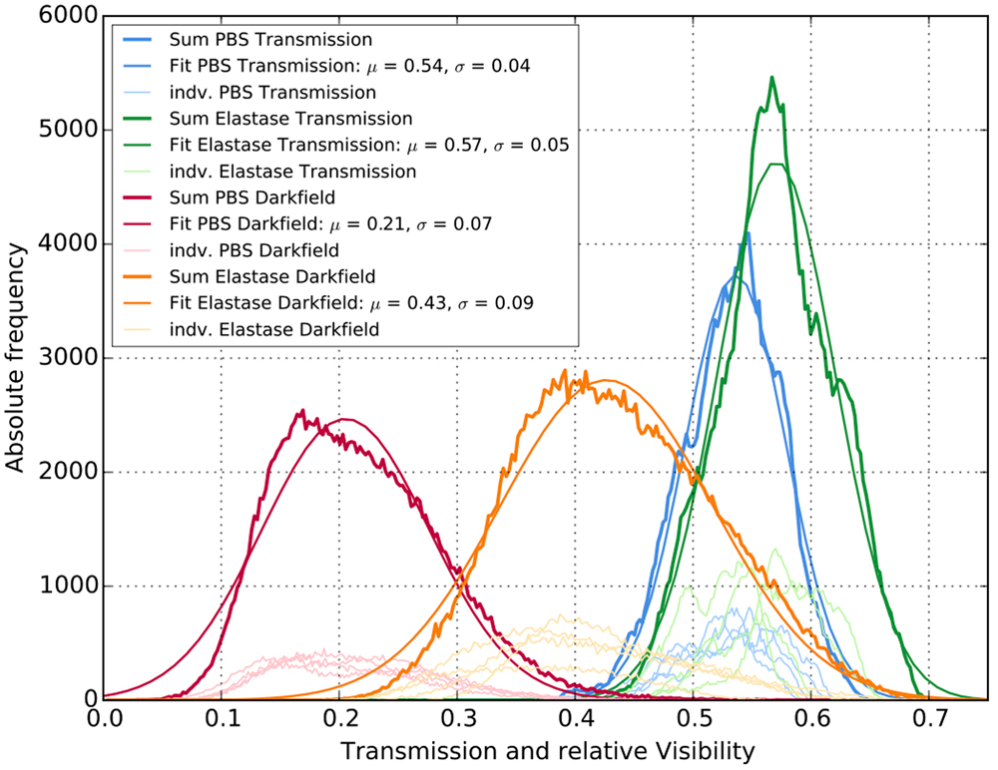
Histogram representation of PBS and elastase treatment comparing attenuation and scatter information at day 1. Individual (light) and group wise summed (dark) signal values extracted of the segmented lung regions. Similar signal values were obtained within the individual groups. A pronounced distribution separation was only observed in the relative visibility signal. Mean (*☐*) and width (*☐*) of the merged distribution for the histogram analysis were obtained by a Gaussian fit.

To investigate the discriminability of the transmission and dark-field signal between healthy and pathological specimen, a basic histogram analysis was performed comparing controls and treated animals at day 1 (Fig. 6). While the signal distributions of PBS and elastase treatment widely overlap in the transmission channel, a distinct separation was obtained for the relative visibility data. Despite its wider distribution, the discriminability between controls and treated animals is 4.12-times higher for the relative visibility compared to the transmission signal.

## Discussion

Acute lung inflammation is a potentially life threatening condition. Early diagnosis is essential to prevent aggravation of this disease. As conventional transmission radiography is neither highly sensitive nor specific concerning lung imaging^20^, making a diagnosis can be challenging. Due to its higher sensitivity dark-field lung imaging might offer a potential solution to this problem.

This study demonstrates for the first time that *in vivo* imaging of acute lung response using dark-field radiography is feasible. Due to the strong dark-field signal derived from healthy lung tissue, inflammation-driven development of acute, bilateral infiltrates, can be clearly visualized, representing a substantial improvement over conventional transmission X-ray imaging.

Transmission images showed subtle, but typical signs of acute lung inflammation, such as diffuse lung opacities. However, when evaluated quantitatively, transmission signal intensities did not alter significantly in acute lung injury. Moreover, transmission images did not conclusively demonstrate the progression of emphysema, which is in agreement with earlier studies^17^. Since lung tissue only weakly absorbs X-rays, changes in lung architecture lead to a minor change in signal intensity, often too weak to be detected visually^21^ or quantitatively. Furthermore, as X-ray beams are attenuated by soft tissue, transmission signal strength, unlike dark-field signal intensity, greatly depends on the overlying chest wall. Hence, transmission signal intensities within the lung parenchyma are always influenced by overlaying structures, complicating the diagnosis of lung diseases. In contrast, it is possible to image acute lung injury, as shown by qualitative and quantitative analysis of the dark-field signal. At day 1, a maximum loss in signal strength can be observed in the dark-field images, indicating the presence of acute lung inflammation. This was confirmed by BAL and histology as lavages showed a substantial increase of leucocytes indicative of inflammation^22^. The observed changes in signal strength are not related to the instillation of mere liquid as one day after application of PBS, in the control group, there was no alteration in signal intensities found, indicating complete resorption of the fluid. 21 days after elastase treatment, as well as in the control groups, neither in BAL nor in histopathology an inflammatory reaction could be observed. This acute rise in the total number of leucocytes in BAL at day 1 after elastase instillation is mainly driven by neutrophils, which is consistent with literature^23,24^. As BAL shows, the count of macrophages in the bronchial spaces is stable. However, histopathology indicated that the quantity of macrophages in the lung alveoli strongly increased. A possible explanation for this observation is most likely based on the fact, that BAL does not reach the smallest, most peripherally located airways where tissue macrophages are located.

MCL quantification highlights, that development of acute lung inflammation as well as emphysema is occurring at the same time: both are measurable one day after application of elastase. This represents a challenge for isolating the effects of each of these processes. Elastase is typically used to induce pulmonary emphysema in mice and therefore is not a specific disease model for lung inflammation. Nonetheless, it is well known that intratracheal instillation of elastase causes an acute lung response, manifesting in alterations of the integrity of the alveolar-capillary barrier, with consequent alveolar flooding, hemorrhage and recruitment of inflammatory cells referred to as diffuse alveolar damage^25^, which we were able to depict with BAL and histology one day after application of elastase. Moreover, correlating the data from quantitative image analysis with both, MCL quantifications and results from BAL, indicates that the alterations in dark-field signal intensity observed at day 1 is not exclusively due to the development of emphysema. Only 1 day after instillation of elastase the development of only mild emphysema can be expected, which cannot be responsible for the immense loss in dark-field signal intensity even exceeding that measured at day 21. The signal development at day 1 is mainly due to changes in acute lung inflammation, such as alveolar edema, minimizing air in the alveoli and therefore leading to a very weak dark-field signal. This is confirmed by the corresponding transmission images. Although the measurable changes in transmission signal intensity might not be significant we clearly find diffuse lung opacities indicating infiltrates caused by application of elastase 1 day prior. The time curve of the measured dark-field signal endorses this assumption. There is a significant dip of the dark-field signal at day 1 compared to the signal strength at day 7 and even at day 21, indicating the presence of a severe infiltrate on top of early pulmonary emphysema. Only seven days after application of elastase, the dark-field signal intensity has significantly increased compared to day 1, which – as pulmonary emphysema is further developing and therefore should lead to an increasing loss of dark-field signal intensity- is explained by a decline in inflammation. As pulmonary emphysema is developing over time, we find a constant reduction of dark-field signal strength from day 7 on until day 21 – as mentioned before – never reaching the level of maximum signal loss observed at day 1, which is mainly due to inflammatory processes in the lungs. However, to definitely exclude the influence of emphysematous changes a disease model specifically designed for lung inflammation should be applied in future studies^26^.

This work is a feasibility study with a small number of animals. Since it was designed as a longitudinal study, histological correlation, proving that the signal changes observed in dark field images at day 7 and 14 represent the real degree of lung destruction, is not available. However, a previous study focusing on grading different stages of emphysema with dark-field radiography showed an excellent correlation between emphysema severity and histology^17^. Therefore, dark-field images acquired at these interim time points can reasonably be assumed to reflect the true degree of lung destruction.

Animals were breathing freely at a respiratory rate of approximately 120 breaths per minute, which might result in some blurring especially in the peripheral regions of the lungs.

Furthermore, a number of technical challenges, such as a larger field of view and further dose reduction (animal dose/acquisition 1–1.5 mGy) need to be solved with regard to a potential clinical application in humans^22^.

To conclude, our study suggests that dark-field radiography allows for significantly more sensitive detection of acute lung inflammation in mice compared to conventional transmission imaging. Combining the high sensitivity provided by dark-field imaging with the rather specific information of transmission images might lead to a maximum efficiency in the diagnosis of acute lung inflammation.

## Materials and Methods

### Small animal protocol

Acute lung inflammation was induced by instillation of 80 μl porcine pancreatic elastase (PPE, Sigma-Aldrich, Munich, Germany) dissolved in sterile phosphate-buffered saline (80 U/kg body weight) in the trachea of pathogen-free female C57BL/6 N mice (Charles River Laboratories, Sulzfeld, Germany, n = 11), aged eight to ten weeks^27^. Endotracheal application of PPE in mice is typically used as a model of pulmonary emphysema, which develops over the course of several weeks. In this study, we used the same mouse model to study the acute lung injury that occurs as an immediate response to the instillation of PPE. Control mice received 80 μl sterile phosphate-buffered saline (n = 10). Animals were imaged immediately before and one day after application of elastase or PBS to assess early changes in pulmonary structure. Mice were breathing freely during image acquisition. Respiratory frequency as well as body temperature was monitored while the pulmonary scans were performed. To protect the animals from hypothermia, a built-in fan kept the temperature inside the scanner constantly warm. Following image acquisition, 6 animals out of each group were sacrificed. By rinsing the lungs 3-times with 0.5 ml sterile PBS using a tracheotomy cannula, bronchoalveolar lavages (BAL) were acquired. Lungs were subsequently fixed by intratracheal instillation of 6% paraformaldehyde for further histological analysis. The remaining mice (n = 5 in the experimental group, n = 4 in the control group) were imaged 7, 14 and 21 days after application of PBS or elastase. At day 21 these animals were sacrificed, BALs were performed as described and the lungs were obtained for histopathological processing. Intraperitoneal injection of midazolam (5 μg/kg body weight), fentanyl (50 μg/kg body weight) and medetomidine (500 μg/kg body weight) served as anesthesia for PBS/elastase application and image acquisition.

All experiments were carried out according to national (Gesellschaft fuer Versuchstierkunde) and international (Federation of Laboratory Animal Science Associations) animal welfare guidelines. The experiments were approved by the local government for the administrative region of Upper Bavaria, Germany.

### Imaging Protocol

All images were acquired in a supine position using a prototype small-animal scanner^28,29^. The scanner’s grating interferometer consists of three gratings: a gold source grating (period (p) = 10 μm, height (h) = 35 μm), a nickel phase grating (p = 3.24 μm, h = 4 μm; distance to source grating 30 cm) and a gold analyzer grating (p = 4.8 μm, h = 45 μm; distance to source grating 45 cm). The scanner was operated with five stepping positions of the source grating. The source ran at 35 kVp and 20 W source power with an exposure time of five seconds per stepping position. The field-of-view was round with a diameter of 5 cm and a spatial resolution of 57 μm (10% MTF). Using Matlab (Mathworks Inc., MA, USA) post-processing of the acquired images was performed.

### Bronchoalveolar Lavage and cell counts

BAL was centrifuged at 400 G for 10 minutes and the resulting cell pellet re-suspended in RPMI-1640 medium (Gibco, Life Technologies, Darmstadt, Germany) for the total cell count using a hemocytometer. Cytospins of the cell suspension were then prepared and stained using May-Grünwald-Giemsa for differential cell counting (200 cells/sample).

### Histology

The right lung was snap frozen in liquid nitrogen for further analysis. The left lung was fixed under a constant pressure of 20 cm by intratracheal instillation of 6% paraformaldehyde and using systematic uniform random sampling embedded into paraffin for Hematoxylin and Eosin (H&E)-stained histological analysis and immunohistochemistry. Images of stained sections were obtained using a Mirax Desk (Carl Zeiss MicroImaging GmbH, Göttingen, Germany) slide scanner and analysed using Pannoramic Viewer version 1.15.2 (3DHistech Kft, Budapest, Hungary).

### Quantitative Morphometry

Using an Olympus BX51 (Olympus, Germany) light microscope equipped with a computer-assisted stereologic toolbox (newCAST, Visiopharm, Hoersholm, Denmark), lungs were analyzed with design-based stereology. Histological evaluation of the lungs was performed using slices of pulmonary tissue that covered the entire cross-section of the right and the left lung in axial plane. Within one slice, 35 randomly selected regions of interest were analyzed with a computer-aided system as specified through published guidelines for MCL-quantification^30^. Mean cord length (MCL) was measured by superimposing a line grid on the images of lung sections at a 20-times magnification. Points on the lines of the grid hitting the air spaces as well as intercepts of the lines with alveolar septa were counted to calculate MCL in μm pursuant to the equation:

$$MCL=\frac{{\rm{\Sigma }}{P}_{air}\,\cdot L(p)}{{\rm{\Sigma }}{I}_{septa}\cdot 0.5}$$

where*P*_*air*_ are the points of the grid hitting air spaces, *L(p)* is the line length per point and *I*_*septa*_ are the intercepts of alveolar septa with grid lines^31^.

### Quantitative Image Analysis and Histogram Analysis

Regions of interest (ROIs) were defined to quantify dark-field and transmission signal intensities in the lung. These pentagonally shaped ROIs were placed manually into the images to capture as much lung tissue as possible. The mediastinal shadows as well as all osseous thoracic structures were excluded. The latter was done to avoid distortion of the transmission signal by the rib cage^17^. For the histogram analysis transmission and relative change of visibility were compared whereas their negative logarithm was used for quantitative comparison with MCL values as both signals scale exponentially with the samples thickness. The dataset utilized for the histogram analysis is depicted in Fig. 6. It consists of the group-wise summed signal values extracted of the aforementioned segmented lung regions. Within the individual groups and modalities similar histograms were obtained as indicated in Fig. 6. For this reason, a merge of the data for the histogram analysis is justified. A Gaussian function was fitted to the data to obtain mean $(\mu )$ and width ($\sigma$) of the respective distributions. The ratio of the mean distances with respect to the distributions width of the respective modalities serves as a performance indicator to compare the two imaging modalities in terms of discriminability between PBS and elastase treatment:

$$\frac{|\frac{{{\rm{\mu }}}_{ELA}-{{\rm{\mu }}}_{PBS}}{{{\rm{\sigma }}}_{ELA}+{{\rm{\sigma }}}_{PBS}}|[Vis]}{|\frac{{{\rm{\mu }}}_{ELA}-{{\rm{\mu }}}_{PBS}}{{{\rm{\sigma }}}_{ELA}+{{\rm{\sigma }}}_{PBS}}|[Tra]}$$

### Statistical analysis

Means and standard deviations of cell counts for mean cord length (MCL) and bronchopulmonary lavage of the control and experimental group were calculated. Means were tested for statistical significance using Student two-tailed t-test for paired samples. Medians and ranges of dark-field and transmission signal intensities derived from the lung tissue were calculated for all animals. Medians were compared between the control and experimental group using Mann-Whitney-test and within one group using Friedman test. MedCalc^®^ (version 15.2.2, Ostend, Belgium) was used as statistical software.

### Data availability statement

All data generated or analyzed during this study are included in this published article.
